# Little Troubles with a Plate Tooth

**Published:** 1857-12

**Authors:** A. Berry


### LITTLE TROUBLES WITH A PLATE TOOTH.

BY A. BERRY, D. D. S.

Some half-dozen years ago I removed a diseased, permanent, mal-formed, superior, central incisor, for a young man about twenty years of age, and inserted another by atmospheric pressure.

A few weeks afterward, he informed me that the plate was not of good gold—that the gold had worn off and left silver exposed. On examination, the lingual surface of it was found nearly the color of silver, but somewhat blackened, while the surface in contact with the mucous membrane presented a good gold color. Instead of using whiting or chalk, as ordered, in cleaning the plate, he had tried a preparation for silver ware, composed of chalk and mercury, "patronized by Her Majesty Queen Victoria, etc." and produced an effect on it that required considerable scraping and friction with pumice stone to remove.

About two years subsequently, the patient called again, with a third tooth through the gum in place of that removed; and after this was extracted, it was necessary to take off the tooth and re-model the plate.

